# Surgical attitude in premalignant lesions and malignant tumors of the lower lip

**Published:** 2015

**Authors:** N Calcaianu, SA Popescu, D Diveica, I Lascar

**Affiliations:** * Clinic of Plastic Surgery and Reconstructive Microsurgery, Bucharest Clinical Emergency Hospital, Bucharest, Romania; ** ”Carol Davila” University of Medicine and Pharmacy, Bucharest, Romania

**Keywords:** lower lip tumor, premalignant lesions, keratoacanthoma, actinic keratosis, squamous cell carcinoma

## Abstract

**Introduction.** Malignant tumors of the lower lip may have a variety of histopathology forms. The diagnosis and treatment of premalignant lesions are extremely important to avoid their malignant evolution. The lower lip tumor diagnosis is based on a series of correlations: anamnestic, clinical, laboratory and histopathological (the latter giving the certain diagnose).

**Material and methods.** This study was carried out by selecting the cases with lower lip tumors operated between January 2012 and July 2014, in the Plastic Surgery and Reconstructive Microsurgery Clinic of Bucharest Clinical Emergency Hospital. The variables considered in the study were the following: age, gender, exposure to risk factors, diagnosis, and histopathology.

**Results.** The histopathological examination revealed 63% squamous cell carcinoma, 30% basal cell carcinomas, 5% keratoacanthoma and 2% actinic keratosis. Men were the predominantly affected genre, with a percentage of 70%. In the group of patients studied, 66% were smokers.

**Discussions.** The rate of the malignant transformation of premalignant lesion was 32.6% for keratoacanthoma, 16.9% for actinic cheilitis, 10% for actinic keratoses.

**Conclusions.** There were no clinical or laboratory features to plead for the pre-malignant or malignant character of the of a lower lip tumor, consequently histopathological examination was used for the diagnosis of the lesion. Due to the high percentage of malignant transformation of precancerous lesions, particularly in the form of squamous cell carcinoma, the surgical attitude intending to eradicate a lower lip tumor from an oncological point of view was the excision with oncologic safety margins followed by a lip reconstruction.

## Background

Malignant tumors of the lower lip may have a variety of histopathology forms. Squamous cell carcinoma is by far the most common type of cancer in this location. Basal cell carcinoma is also common, located in the skin of the lip. Mucosal melanoma, especially in the oral cavity, is rare, but is more aggressive and has a poorer prognosis. Malignant tumors of the small salivary glands may appear on the oral face of the lip and rarely, malignant tumors can appear on the lips developing from other structures: leiomyosarcoma, fibrosarcoma, lymphoma, angiosarcoma, rhabdomyosarcoma. While the incidence of lip cancers in the central Europe is low, - 0.7% of all malignant tumors compared to the 1-2% generally considered [**1**-**4**], they are extremely important from the clinical and surgical point of view because of the morphological and functional changes involved. The management of these lesions is complex and depends on a number of factors: the type of tumor and metastasis possibilities, ablation techniques, reconstruction possibilities and adjuvant treatment. Squamous cell carcinoma frequently occurs as a premalignant lesion: actinic keratosis, chronic cheilitis, leukoplakia or keratoacanthoma and, in 90% of cases, the onset is the in the lower lip mucosa. Diagnosis and treatment of premalignant lesions is extremely important to avoid their evolution to malignancies. The factors responsible for producing carcinomas are the following: UV radiation, smoking, chronic trauma, preexisting lesions with malignant transformation potential, viruses (human papillomavirus, retroviruses), the existence of immunosuppression [**8**-**9**]. Men are more at risk than women, (1.3% men vs. 0.3% women) [**1**,**5**-**8**]. In its The tumor, in its initial phase, usually appears as a papule or a plate which tends to progress into a vegetative or ulcerative form. The evolution of squamous cell carcinoma is more aggressive than basal cell carcinoma because it has a rapid growth rate with increased local invasiveness and with high capacity of metastasis. Lower lip tumor diagnosis is based on a series of anamnestic, clinical, laboratory and histopathological correlations. A correct assessment of the lesion is very important for a correct excision strategy aiming the oncologic resection of the tumor without recurrences.

## Materials and methods

This study was carried out by taking cases of lower lip tumors operated between January 2012 and July 2014 in the Plastic Surgery and Reconstructive Microsurgery Clinic of Bucharest Clinical Emergency Hospital. The number of patients was 42. The variables considered in the study were the following: age, gender, exposure to risk factors, diagnosis, and histopathology examination. The surgical attitude consisted in the excision of the tumor formation within the oncologic safety limits, considered to be of 7 mm to 1 cm from the macroscopic tumor margins and the reconstruction of the lower lip by using the Camille-Bernard modified technique. All the patients underwent preoperative screening examination for the possible matastasis: abdominal echography, lungs radiography, cerebral CT (when possible). The aim of this study was to determinate the rate of the premalignant lesions which had an anamnestic rate of evolution, macroscopic features and clinical signs similar to the malignant lesion cases by using the histopathology examination results, and thus to assess the correct surgical decision for a complete tumor ablation, reducing the possibilities of an unfavorable evolution of the lower lip lesion.

## Results

The histopathological examination revealed a 63% squamous cell carcinoma, 30% basal cell carcinomas, 5% keratoacanthoma and 2% actinic keratosis.

**Fig. 1:**
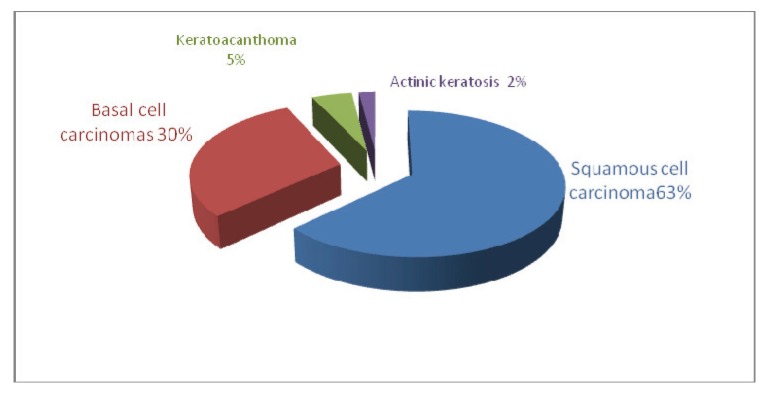
The classification of tumors according to the histopathological diagnosis

Men were the predominantly affected genre, with a percentage of 70%.

**Fig. 2:**
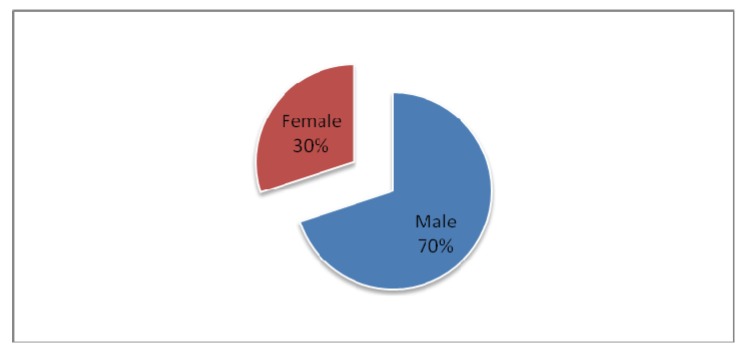
Distribution of patients according to gender

In the group of studied patients, 66% of them were smokers, the result being the role of tobacco as a risk factor in the development of tumors of the lower lip.

**Fig. 3:**
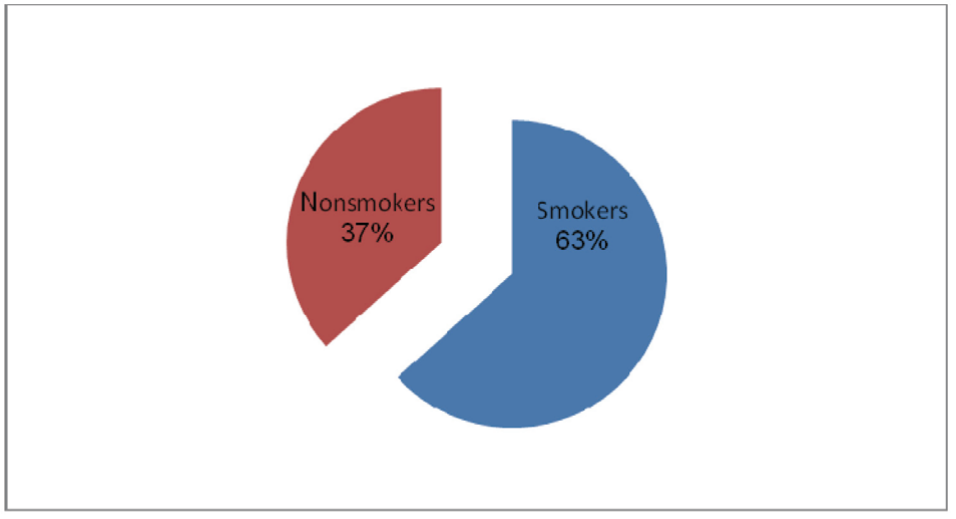
Distribution of patients according to the smoking habit

In our study group of patients, there were 17 excisions followed by a direct suture, the rest of the cases being operated with a modified Camille Bernard technique. Lymph adenectomy was performed in the same time with the lip reconstruction in four patients. In all the other cases with a confirmed spinocellular histology examination, lymph adenectomy was done a month to two months after the local block excision of the tumor followed by the lip reconstruction.

## Discussions

The rate of the malignant transformation of premalignant lesion was 32.6% for keratoacanthoma [**12**], 16.9% for actinic cheilitis [**13**], 10% for actinic keratoses [**14**,**15**]. Histopathology examination should be used to confirm the diagnosis of epithelial precancers, because, often, the clinical appearance of premalignant lesions is very similar to a histopathology confirmed squamous cell carcinoma. Keratoacanthoma has a trend of persistence and progression to invasive squamous cell carcinoma. The highest risk of transformation for actinic keratoses is for the ulcerated lesions and hyperkeratosis. The emergence of malignancies was positively correlated with age, with the infiltration of the lesion, the occurrence of pain (in 50% of the cases this correlating with malignancy) and the size of the lesion.

Due to the high percentage of malignant transformation of precancerous lesions, especially to a squamous cell carcinoma, the attitude should be surgical excision in oncological safety margins followed by the reconstruction of the lower lip. The surgical techniques used to reconstruct post excision defects depended on the size of the defect. The main reconstruction technique for the defects of the lower lip, larger than 2/3 by using tissues from the neighboring regions was the Camille-Bernard modified technique. This technique consists of a complete excision of the lower lip practicing chin tegument incisions in “W” or incisions “in barrel” that may extend horizontally outside the commissure, then two triangles are excised all over the thickness of the sides of the upper lip to advance medial two nasolabial flaps [**10**]. The Camille-Bernard technique allows the restoration of the commissure does not cause microstomia and allows the easily rebuilding of the vermilion with mucosa [**11**].

The malignant tumors of the lower lip are characterized by a locally invasive potential and a higher incidence of metastasis. Although the stage T1 or T2 percentage of patients with lymph node metastasis at the time of diagnosis was 8%, this figure rises considerably in advanced tumors, making it necessary to search for possible metastatic cervical lymph nodes [**2**]. Lymphatic metastasis usually occurred after the primary tumor was identified. The time of lymph node metastasis is frequently related to the aggressiveness of the primary tumor, its size and its differentiation. However, the size of the primary tumor is not necessarily associated with the degree of regional metastasis. In the oncology context, the absence of palpable cervical lymph nodes shows no metastasis, small reagents lymph nodes or metastatic microangiopathy.

Depending on the time of the cervical lymph node excision, this can be done in one surgery operation, the advantage being the removal of the primary tumor and cervical structures in one-piece. Preferably, cervical neck dissection can be performed 4 weeks after the surgery due to the following advantages: usually, less surgical time on old patients avoids the association of a septic time (oral) with an aseptic time (cervical). Also the lymph nodes act as a filter for tumor clones disseminated during surgery and at four weeks after surgery we have the confirmation of a malignant or premalignant lesion after the histopathology examination.

## Conclusions

There are no clinical or laboratory features to plead for the pre-malignant or malignant character of a lower lip tumor, consequently histopathology examination is used for the diagnosis of the lesion, especially because often, the clinical appearance of a premalignant lesion corresponds to the appearance of a histopathology confirmed squamous cell carcinoma. Due to the high percentage of the malignant transformation of precancerous lesions, particularly in the form of squamous cell carcinoma, the surgical attitude should be the excision of the lower lip tumor in oncological safety margins followed by the reconstruction of the lip.
